# Evaluating the drivers of B2B performance: An empirical analysis based on Alibaba

**DOI:** 10.1371/journal.pone.0306919

**Published:** 2024-07-12

**Authors:** Miao Feng, Haoran Si, Yang Li, Junrui Zhang

**Affiliations:** 1 Business School, Shandong Management University, Jinan, China; 2 Business School, Shandong Normal University, Jinan, China; King Khalid University, SAUDI ARABIA

## Abstract

The rapid development of B2B has brought about fierce competition among suppliers, and how to gain customer attention and improve performance has become a common concern in academia and industry. This study examined the drivers and mechanisms of B2B performance from an enterprise capability perspective. We collected transaction and enterprise data from 325 suppliers on Alibaba 1688 platform and constructed a structural equation model (SEM). Results showed that supplier service capability, logistics capability, and production capacity all positively impacted B2B performance through the mediating role of customer attention. In addition, we found that service and logistics capabilities are more critical for attracting customer attention for Original Equipment Manufacturer (OEM) suppliers than for non-OEM suppliers. The findings contribute to understanding B2B commerce and provide constructive directions for B2B suppliers to improve their performance.

## Introduction

B2B e-commerce is an Internet-based transaction facilitator that meets the transactional needs of buyers and sellers while providing value-added services [1]. B2B e-commerce has become the dominant mode of business transactions because it has subverted the traditional multi-tiered supply chain relationships, made the market more open and accessible, and created considerable economic benefits [2]. In 2015, Amazon began to push into the B2B space, changing the name of its B2B e-commerce business from Amazon Supply to Amazon Business, with sales of more than $1 billion in just one year. By 2022, the registered Gross Merchandise Volume (GMV) has reached $35 billion. Alibaba, the world’s largest B2B platform, now has suppliers from more than 190 countries and more than 100 million active business buyers.

However, B2B e-commerce can be a double-edged sword, increasing transaction efficiency while intensifying market competition. At the customer acquisition stage, due to factors such as the complexity of downstream customer types, it is difficult for B2B suppliers to accurately analyze downstream buyers’ business scenarios and stimulate buyers’ purchasing decisions through accurate and high-quality information disclosure [3,4]. In B2B business platforms, when transaction records and business information between enterprises become visible, potential buyers can scrutinize suppliers’ qualifications and make purchasing decisions based on this information [5]. Therefore, suppliers’ capabilities demonstrated in previous transactions are critical to attracting customers [6]. What exactly are the capabilities that can attract customer attention to improve business performance?

B2B commerce research has received increasing academic attention, with many scholars focusing on the factors influencing B2B performance [7]. The existing literature can be divided into two streams, one of which suggests that B2B performance is affected by upstream and downstream relationships in the supply chain, with many studies proving that reciprocity [8], trust [9], and relational commitment [10] contribute to B2B performance. The other stream posits that in the B2B business environment, enterprises’ business strategy is market-oriented, and competitive intelligence in the network is an essential basis for deal-making, implying that B2B performance can be driven by supplier capabilities [5]. It was found that market skills, competencies, and customer contact capabilities to fulfill customer requirements can improve B2B performance [11]. In addition to the supplier capabilities listed above, studies have shown that brand capability [12], innovation capability [13], and organizational capability [14] can influence B2B performance.

However, there is still a shortage of research addressing the capability perspective of the B2B supply chain. From the perspective of Porter’s value chain model, most studies focus on supporting activities, and research on primary activities is far from comprehensive, encompassing only marketing capabilities, while the other three essential capabilities, logistics capabilities, service capabilities, and operation capabilities, have been overlooked. In addition, there are different types of suppliers on B2B platforms, especially OEM suppliers, which are significantly different from non-OEM suppliers in terms of their capabilities [15]. Since downstream buyers may have different initial attitudes to whether a supplier is an OEM, there may be discrepancies in the capabilities that different suppliers need to disclose to improve B2B performance. However, existing studies do not distinguish between these two types of suppliers. Addressing this issue could contribute to identifying the boundaries of the impact of supplier capabilities on B2B performance and allow suppliers to allocate their resources more rationally and efficiently.

Therefore, this study aims to fill the gap in the drivers of B2B performance by comprehensively examining the impact of service, production, and logistics capabilities on B2B performance and testing the mediating role of customer attention in the process. Moreover, we innovatively distinguished the suppliers into OEM and non-OEM manufacturers and explored the capability diversity required by different types of suppliers to enhance B2B performance. This study contributes to the theoretical system of B2B performance research by revealing the impact of supplier capabilities on B2B performance and discovering the mechanisms and theoretical boundaries of this impact. The findings will help B2B suppliers deepen their understanding of the cooperation mechanism between enterprises and target their capabilities to achieve efficiency gains.

To advance this line of research, we targeted Alibaba’s 1688 trading platform and collected transaction data and capability information from 325 suppliers, of which the capability information includes service capability, production capacity, and logistics capability, and explored the impacts of these three capabilities on B2B performance; furthermore, we examined the mediating role of customer attention in these impacts and the moderating role of OEM certification in these impacts. For future research, we suggest that more market factors should be considered, and mathematical models could be used to investigate the impact of suppliers’ strategic behavior or market competition on B2B performance.

This paper is structured as follows: We first present the theoretical background and the formulation of the hypotheses. Next, we describe the research methodology and the results of the empirical analyses. Finally, we discuss our main findings, theoretical contributions, managerial implications, and limitations.

## Literature review

### B2B commerce

B2B electronic commerce refers to the mode of business-to-business transactions centered on information technology, and its large-scale commercial application can be traced back to electronic data interchange (EDI) in the 1980s [16]. With the spread of the internet, B2B e-commerce has evolved into more open and flexible forms, including online marketplaces, e-procurement platforms, and supply chain management systems. Using platforms is strategically vital to buyers because it can help companies break time and space constraints to improve the efficiency of inter-enterprise transactions and reduce the transaction costs associated with the supply chain [17]. Low transaction costs have also attracted more sellers and buyers to participate in B2B platforms, which have become more efficient because of network externalities [18]. In addition to reducing transaction costs, the intervention of B2B platforms can also effectively realize digital marketing at the industry level, acquire good customer word-of-mouth and customer value, and, thus, increase the number of transactions and business performance [19,20].

In the early stage of the B2B business platform launch, it is critical to design the platform’s mechanics to engage potential buyers and ensure they stay on the platform for the long term. Factors affecting B2B customer adoption can be categorized into product factors (e.g., product specificity, product value), degree of market change, and individual factors (frequency of purchases, IT capabilities, and efficiency motives) [21,22]. When customers enter the B2B e-commerce platform, the effective operation of the platform becomes a vital issue. Many scholars have studied the construction of trust and cooperation among participants in B2B e-commerce platforms. Institutional trust in B2B e-commerce platforms is a prerequisite for promoting customer inter-organizational trust. Mallapragada et al. [23] investigated the effect of virtual inter-organizational relationships on user trust and satisfaction regarding interdependence and relative dependence; they found that relative reliance only significantly impacts new interuser relationships, and this effect diminishes over time. On the platform, when there is interdependence and relative dependence in the relationships between old users, their satisfaction increases. Nevertheless, the trust and satisfaction of new users decreases.

Platform governance is a vital research topic in the development of B2B commerce. Unlike traditional B2B relationships, B2B e-commerce platform governance studies the management of B2B platform enterprises over bilateral enterprises. When considering the contribution behaviors performed by multiple participants on a B2B platform, the effects of learning and competition must be noted [24]. Grewal et al. [25] devised three approaches to the governance of platform enterprises: monitoring, community building, and autonomous platform participation; they considered the roles played by the three governance approaches in the context of platform reputation, pricing approach, and demand uncertainty conditions and found that monitoring is the best form of platform governance when the platform reputation is high, and market demand is uncertain. When the pricing model is static rather than dynamic, community building is the best mode of platform governance. Self-participation is the best platform governance approach when the platform’s reputation is high, and pricing is dynamic.

### B2B commerce performance

B2B commerce performance is the extent to which an e-marketplace delivers and enhances value to its owners and how efficiently it performs its tasks and achieves its goals [26]. Many scholars have used financial metrics, such as transaction volume, to measure good or bad B2B performance [27]. In addition to financial indicators, Wang et al. (2012) used the number of enterprises participating in the market as a performance measure, arguing that the more enterprises participate in the B2B market, the larger the size of the B2B market, the stronger its network externalities, and the greater the market value it can create [28]. Matook (2013), on the other hand, used 16 indicators to comprehensively measure B2B market performance, including transaction volume, customer loyalty, and the number of buyers [29].

How to effectively improve B2B market performance is a hot topic in academia and industry in the B2B field. Many scholars have explored the influences that affect B2B performance from the perspective of organizational resources and capabilities. Wang et al. suggested that online marketing, online social networking, product/service quality, and learning capabilities are essential drivers of B2B performance [28]. Thitimajshima et al. (2017) explored the factors affecting B2B market performance from multiple perspectives, such as business relationships, transactions, and marketplace services. They found that e-marketplaces, trusting relationships between merchants, transaction cost reductions, and website usability can significantly affect loyalty, website reliability, and relative advantage and that several buyers considerably impact B2B performance [30]. Previous research has explored the drivers of B2B performance from the perspectives of relationships and online marketing capabilities but neglected that downstream buyers first need to examine and filter the qualifications of upstream suppliers before concluding B2B transactions. Therefore, the current study fits in to fill this gap by exploring the impact of suppliers’ capability information on B2B market performance.

### Single theory

Signal theory, which was first proposed by Michael Spence, was initially used to address the problem of information asymmetry in the labor market and mainly consists of the sending of signals, the interpretation of signals, and the feedback of signals [31]. In a B2B context, the features or information displayed on the platform can be regarded as signals from the seller to the buyer. Buyers view and interpret signals and engage in market behaviors [32]. However, product and seller information is not entirely open and transparent, so there is a general problem of information asymmetry between online buyers and sellers [33].

Most studies have focused on the effect of the type and number of signals on both sides of a transaction [34]. A survey by Mavlanova et al. (2012) revealed differences in signal selection between low- and high-quality sellers in the marketplace. The present study revealed that sellers tend not to act on signals in isolation but rather to use a variety of signals in combination. They identified two signal combination strategies: a combination of high-cost but easily verifiable signals and low-cost but difficult-to-verify signals [35]. High-quality sellers are more inclined to use high-cost signals, including high-quality customer service, product warranties, or professional certifications. Sellers can communicate their reliability and value to the marketplace by choosing these signals and increasing consumer trust and loyalty. In contrast, low-quality sellers use less costly, challenging signals to verify, including false advertising, selling at low prices, and hiding real quality issues. Despite the low cost of these signals, they tend to hurt the market, reduce consumer trust and may lead to market instability or imbalance. Mavlanova et al. (2016) studied the effect of external and internal signals on buyers’ perceived seller quality and found that external signals had a significant effect on perceived seller quality, while the impact of internal signals was not substantial. Therefore, to better engage the audience, sellers are advised to provide more external signals [36]. Our study combines signaling theory to investigate what kind of signals merchants provide buyers regarding transaction volume, the return rate, the number of buyers, the number of intended purchases, and the impact on buyers’ perceptions.

## Research hypothesis

### B2B supplier capabilities and performance

Production capacity is a capability resource that integrates tangible and intangible manufacturing resources; it includes several production and operational metrics, such as production volume, resource utilization, and flexibility, which can directly impact supplier performance [37]. In a B2B environment, production capacity is an important metric, and downstream enterprises usually assess the production capacity of their suppliers through data, such as the amount of equipment, production lines, and factories [38]. The richer a B2B supplier’s capacity information is and the stronger the production capability it possesses, the more likely a potential customer is to perceive the supplier as having better supply chain stability, flexibility, and operational capability and as being better able to cope with unknown risks and guarantee on-time delivery of the product or service and therefore more willing to transact with that supplier.

Transactions in e-commerce contexts are more transparent and visible, and downstream buyers can observe the performance of upstream suppliers or sellers on the service side, for example, through online reviews [20]. In a B2B environment, a supplier’s service capability can directly impact customer satisfaction, financial performance, and the relationship between the buyer and seller [39]. For B2B suppliers, their service capabilities help increase the attractiveness of cooperation, reduce the perceived risk to customers, and make downstream companies more confident in establishing partnerships with them by establishing good communication channels and providing customized solutions.

Rapid and efficient logistics services are critical for enterprises to gain market share [40]. In B2B platforms, logistics service quality is mainly reflected in service effectiveness and reliability [41]. From the timeliness perspective, logistics service quality is primarily reflected in logistics speed, information transfer quality, order processing time, and other factors. The timeliness of logistics can accelerate an enterprise’s capital turnover to improve its performance [42]. In addition, at the logistics service level, downstream customers attach great importance to logistics service reliability, which can be defined as the ability of upstream customers to guarantee timely delivery more adequately [43]. Therefore, superior logistics capabilities can increase customer reliability, facilitate transaction closure, and enhance B2B platform performance. Hence, we propose the following hypothesis:

*H1a*: *Production capabilities of suppliers positively impact B2B performance*.
*H1b*: *Service capabilities of suppliers positively impact B2B performance*.
*H1c*: *Logistics capabilities of suppliers positively impact B2B performance*.

### Mediating effects of customer attention

Attention is a vital resource for firms that can impact many factors, including product promotion and market performance [44,45]. However, because of the limited ability of individuals to pay attention, there is competition among enterprises for customer attention [46]. Many B2B platforms have developed social network features that allow downstream customers to follow upstream suppliers. When potential buyers notice that a seller is getting more attention, they perceive that seller as more reliable and are more willing to enter into a transaction with the seller [11]. Considering the information asymmetry problem in B2B platforms, potential buyers are often at an information disadvantage because of cognitive limitations or intentional concealment by sellers. The attention of more buyers can be seen as a signal provided by the market, which can reduce the perceived risk of buyers and facilitate the finalization of a deal [47]. Therefore, the current study argues that customer attention mediates supplier capabilities and B2B performance. For example, the stronger the supplier’s capabilities are, the easier it is to attract buyers’ attention, ultimately boosting B2B performance. Based on the above analyses, the current study proposes the following hypotheses:

*H2a*: *Customer attention mediates the effect of B2B suppliers’ production capabilities on their performance*.
*H2b*: *Customer attention mediates the effect of B2B suppliers’ service capabilities on their performance*.
*H2c*: *Customer attention mediates the effect of B2B suppliers’ logistics capabilities on their performance*.

### Moderating effects of OEM status

The most significant difference between OEMs and non-OEMs is their production capacity. A superior OEM must have a large enough production capacity to produce and process the products required for brand owners [48]. OEMs are usually responsible for assembly and custom processing, integrating parts and components into a final product [49]. They are more dependent on external suppliers for standard components and raw materials, with the possibility of outsourcing production and commissioning specialized manufacturers to produce components. This makes the OEM production process more flexible and adaptable to the customization needs of its customers [14]. On B2B websites, the OEM status can be seen as the brand owner’s endorsement of the factory, establishing an initial favorable impression and trust for potential customers [50]. When OEMs further disclose more information about their production capacity, services, and logistics, they can create a confirmation effect that makes customers more determined to cooperate with them, thus enhancing their performance. From another perspective, OEM manufacturers are more substantial and usually receive large orders, and they need to collaborate with brand owners for part of the product development work, which mainly focuses on the selection/procuring of raw materials and recommending and confirming processing techniques. However, other potential buyers may be concerned about whether the OEM manufacturer has sufficient spare capacity to provide services. Therefore, the OEM supplier needs to demonstrate adequate production, service, and logistics capabilities to convince potential buyers that they have enough production capacity to secure the completion of production tasks and to realize the timely delivery of products. Therefore, the following hypotheses were formulated for the current study:

*H3a*: *The impact of production capacity on customer attention is significantly greater for OEM suppliers than for non-OEM suppliers*.
*H3b*: *The impact of service capability on customer attention is significantly greater for OEM suppliers than for non-OEM suppliers*.
*H3c*: *The impact of logistics capabilities on customer attention is significantly greater for OEM suppliers than for non-OEM suppliers*.

**Fig 1:**
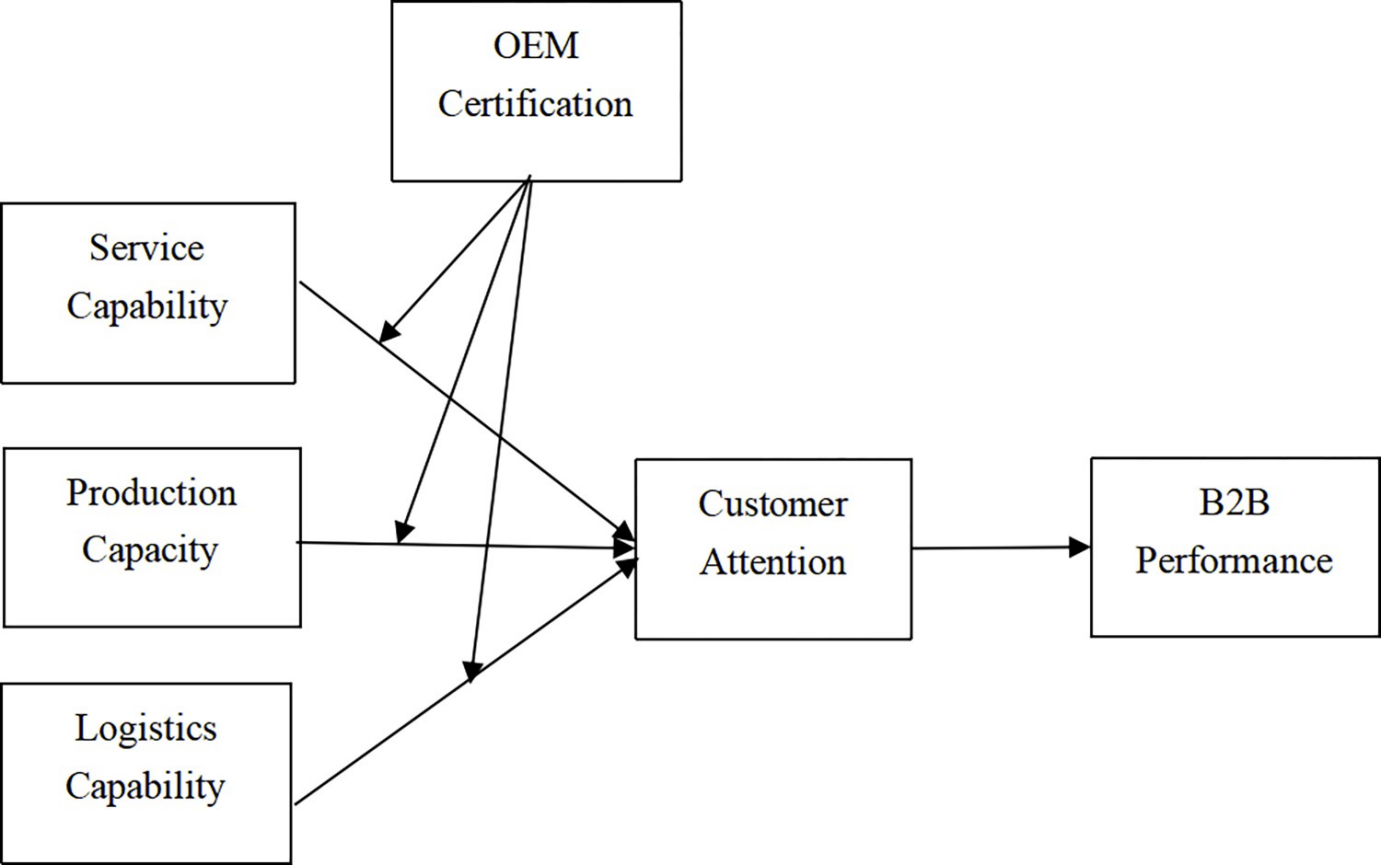
Conceptual model.

The conceptual model for this study is summarised in Fig 1.

## Methodology

### Research data

The data come from Alibaba 1688, which was founded in 1999 and is now the largest integrated B2B trading platform in China, providing matching and online trading services between origin factories, wholesaler sellers, and wholesaler buyers in the areas of apparel and jewelry, packaging materials, office supplies, home decoration and building materials, and digital computers. The website has more than 10 million active customers from 190 countries and regions. The site allows B2B sellers to disclose information, such as company profile and production capacity. In addition, the service performance and performance of B2B sellers are publicly visible on this site.

The present study has chosen daily use products as the research object because these products have high sales on the website and are representative. The specific data mainly include B2B supplier profiles, B2B sales, potential customer attention, supplier response rate, delivery time, number of devices, business registration time, and breach of trust records.

### Variables

The dependent variable in the current study is B2B seller performance, as measured by the cumulative sales of B2B sellers. The independent variables include B2B supplier service capability, logistics capability, and production capacity, where service capability is measured by the customer service response rate, logistics capability is measured by product transit time, and production capacity is measured by the number of production lines owned by the supplier. The mediating variable is customer attention, expressed as this supplier’s amount of customer attention. The control variables in the current study include the enterprise’s age, which is measured as the duration of the enterprise’s registration. The credit of enterprises is a dummy variable, which is expressed by whether the company has a breach of trust, with 1 being the presence of a violation of trust and 0 being the absence of a breach of trust. The descriptive statistics and correlations of the variables are shown in Table 1.

**Table 1:** The descriptive statistics and correlations of the variables.

Variables	Measures	Mean	SD	Max.	Min.
B2B performance	B2B supplier product sales	1817.735	4514.355	38627.000	17.000
Service capability	Customer service response rate	0.819	0.087	0.990	0.670
Logistics capability	Delivery time (week)	22.298	22.160	160.800	2.400
Production capability	The amount of equipment owned by the supplier	51.385	73.990	466.000	1.000
Customer attention	Number of potential customers’ attention	155.191	907.420	3175.000	6.000
OEM	Dummy variable, 1 for OEM suppliers and 0 for non-OEM suppliers	0.271	0.444	1.000	0.000
Enterprise age	Establishment years of B2B suppliers	7.978	4.456	24.000	1.000

### Data analysis and results

Before conducting the hypothesis testing of the structural equation model (SEM), we used the variance inflation factor (VIF) to check for multicollinearity, and the VIF of each variable was less than 2, indicating that the model did not have a multicollinearity problem. Next, the model fit goodness-of-fit metrics are shown in Table 2, and the results all indicate that the model possesses a good fit.

**Table 2:** The model fit goodness-of-fit metrics.

Model fit index	Observed value	Threshold value
SRMR	0.033	<0.08
NFI	0.956	>0.9
RMS Theta	0.098	<0.12

Source(s): Hu and Bentler [51]; Bentler and Bonett [52]; Lohmoller [53].

The importance of validity and significance testing of parameter estimates was emphasized [37,54], and to achieve practical path analysis, SMART PLS 4.0 software was used; the results are shown in Table 3. The present study controlled for enterprise age and business credit when testing for main effects. The main effects analysis showed that B2B suppliers’ service capability, logistics capability, and production capacity all had a significant positive impact on customer attention (β = 0.201, p < 0.01, β = 0.114, p < 0.01, and β = 0.111, p < 0.05, respectively), and hypotheses H1a, H1b, and H1c were supported. These results suggest that information about suppliers’ capabilities is crucial in B2B commerce platforms; when suppliers’ service, logistics, and production capabilities are more vital, they can gain more attention. In addition, customer attention has a significant positive effect on B2B performance (β = 0.739, p < 0.01), and the more attention a B2B supplier receives in the early period, the greater the B2B performance is; thus, hypothesis H2 is supported. Existing studies have confirmed the critical role of customer attention in online communities and online marketplaces, and this finding reaffirms the significance of customers in the B2B business marketplace.

**Table 3:** The results of the path analysis derived from Smart PLS 4.0.

Hypotheses	Path	β	t value	Results
Direct Path				
H1a	Service capability→Customer attention	0.201	4.449***	Supported
H1b	Logistics capability→Customer attention	0.114	3.012***	Supported
H1c	Production capability→Customer attention	0.111	2.236**	Supported
H2	Customer attention→B2B performance	0.739	8.429***	Supported
Mediation Path				
H2a	Service capability→Customer attention→B2B performance	0.148	3.886***	Supported
H2b	Logistics capability→Customer attention→B2B performance	0.084	2.698***	Supported
H2c	Production capability→Customer attention→B2B performance	0.082	2.414**	Supported
Moderation Path				
H3a	OEM*Service capability→Customer attention	0.238	3.619***	Supported
H3b	OEM*Logistics capability→Customer attention	0.155	2.670**	Supported
H3c	OEM*Production capability→Customer attention	0.136	1.837*	Not Supported

Notes

* p<0.1

**p<0.05

***p<0.01.

To conduct the mediation effect test, we refer to Rungtusanatham et al. [55] and use bias-corrected bootstrapping to verify the significance of the indirect effect. The bootstrapping results are based on 5000 bootstrapping samples. We first test hypothesis H2a, the mediating role of customer attention in the impact of B2B supplier service capability on B2B performance. The indirect effect of service capability on B2B performance was found to be significant and positive (β = 0.148, p < 0.01), with a bias-corrected 95% confidence interval [0.072, 0.222], and hypothesis H2a was supported. The indirect effect of logistics capability on B2B performance was significant and positive (β = 0.084, p < 0.01), with a bias-corrected 95% confidence interval [0.036, 0.153], and hypothesis H2b was supported. Finally, the indirect effect of production capacity on B2B performance was significant and positive (β = 0.082, p < 0.01), with a bias-corrected 95% confidence interval [0.016, 0.150], and hypothesis H2c was supported. These findings further suggest that service, logistics, and production information from B2B suppliers can trigger customers’ attention as an essential market signal. Such attention can help reduce the perceived risk and uncertainty of potential buys and enhance the future sales performance of B2B suppliers.

Regarding the moderating effect, the moderating effect of OEM on the relationship between the impact of service capability and B2B performance was significant and positive (β = 0.176, p < 0.01), suggesting that the service capability of OEM suppliers contributes more to customer attention than that of non-OEM suppliers, and Hypothesis H3a was verified. The moderating effect of OEM on the relationship between the impact of logistics capability and B2B performance was significant and positive (β = 0.115, p < 0.01), suggesting that the logistics service capability of OEM suppliers contributes more to customer attention than that of non-OEM suppliers, and Hypothesis H3b was verified. However, the moderating effect of OEM on the relationship between the impact of production capacity and B2B performance was not significant (β = 0.101, p < 0.05), suggesting that for OEMs and non-OEMs, the paths of the effect of production capacity on B2B performance are not significantly different.

## Discussion

### Conclusion

Focusing on B2B performance issues, the current study explored the impact of supplier capabilities, such as B2B upstream suppliers’ service, logistics, and production capabilities. In addition, based on signaling theory, the current study further tested the mediating role of downstream customer attention in the impact of supplier capabilities on B2B performance and the moderating role of the seller’s OEM status. Specifically, we drew the following conclusions:

First, a B2B supplier’s service, logistics, and production capabilities all positively impact its performance. This finding is consistent with existing studies that have explored the relationship between service capability and B2B performance. Moreover, we highlighted the importance of the production capacity and logistics capability of B2B upstream suppliers, in addition to their service capability. With the rise of new business formats, such as live-streaming e-commerce, downstream customers have a greater demand for quick responses from upstream suppliers. Customers need to be sure that suppliers have sufficient production capacity before entering into a transaction and can guarantee the timely arrival of goods.

Second, we found that the attention of potential buyers mediated the relationship between supplier capability and B2B performance. The visibility of B2B platforms allows buyers to observe each other’s behavior. The information cascade or herd effect that is prevalent in individual social networks also exists in B2B networks: The more robust the supplier’s service, production, and logistical capabilities are, the more it can attract the attention of more potential downstream customers, and this high level of attention also leads to faster cooperation with the supplier and higher levels of performance.

Third, OEM suppliers work with specific brands or enterprises to produce products according to their specifications and designs, hence having high production standards in the production chain. However, our research further revealed that improving their service and logistics capabilities is equally crucial for OEM suppliers to attract customers and increase sales.

### Theoretical contributions

The present study makes two critical theoretical contributions. First, we provide a new research perspective for the study of B2B seller performance, as existing studies have focused mainly on the impact of corporate service capability and B2B network structure on B2B performance. Based on B2B supplier capability, the current study extended the value performance of B2B performance to logistics capability and production capability, proving that suppliers can improve their corporate reputation and attract consumers to buy by spreading the three significant signals of their own service capability, logistics capability, and production capability, which are ultimately manifested in B2B performance. Second, the current study enriches the theoretical system of B2B performance research and further reveals the mechanisms and boundaries of the influence of supplier capability on supplier performance. Although most of the existing research on B2B supply chains has been conducted from a trust and relationship perspective, we provide a new perspective on customer focus that offers a new direction for future research.

### Practical implications

The current study offers valuable insights into the management practices of B2B enterprises. In the contemporary digital landscape, enterprises can integrate the enhancement of critical capabilities, such as service, logistics, and production capabilities. B2B platforms should leverage these insights to design features highlighting supplier capabilities and fostering customer attention. Many B2B platforms encourage enterprises to establish a matrix of integrated service offerings, including store construction, marketing, trading, customer management, and after-sales consulting, to provide enterprises with business-critical operational support and to achieve the core objectives of improving transaction efficiency and driving traffic conversion. Moreover, by leveraging AI and big data technology, the enterprise could empower transaction parties with digital intelligence across dimensions, such as business analysis, marketing customer acquisition, and resource matching. These initiatives can accelerate enterprise digital transformation and upgrading, enhance industrial-end business quality, and aid platforms in bolstering brand power to attract a broader customer base. Finally, we recommend that OEMs adopt a differentiation strategy in terms of service and logistics, strengthening their business level and combining it with word-of-mouth promotion to increase user attention. In terms of production, they should continuously improve their flexible manufacturing capabilities to guarantee high production levels.

### Limitations and future research

Our work has some inevitable limitations, but all of these points point to directions for future research. First, the current study adopted specific independent variables (service, logistics, and production capability) for the analysis; however, other vital factors that may impact B2B sellers’ performance, such as market competition and supplier behavior. Due to the limitations of the research methodology, these factors cannot be effectively identified and examined through empirical analysis. We suggest that future research could build on existing studies and use mathematical models to investigate the impact of supply chain competitive relationships and dynamic supplier behavior on B2B performance [56,57]. Second, although whether the supplier is an OEM is introduced as a moderating factor, the differential impact on firms of different industries and sizes has not been analyzed in depth. Further research could extend our study by exploring the underlying mechanisms of how OEM certification affects customer attention and B2B performance and by examining additional industry and firm characteristics.

## Supporting information

S1 Data (CSV)

S1 File (DOCX)
