# Supplementary material for: Evaluating the drivers of B2B performance: An empirical analysis based on Alibaba
Source: PLoS One. 2024 Jul 12;19(7):e0306919. doi: 10.1371/journal.pone.0306919 (PMC11244795; doi:10.1371/journal.pone.0306919)
Supplement: S1 File — (DOCX) [file pone.0306919.s002.docx]

**Author Contributions**

Conceptualization: Miao Feng, Yang Li.

Data curation: Miao Feng, Haoran Si.

Formal analysis: Haoran Si, Yang Li.

Investigation: Haoran Si, Junrui Zhang.

Methodology: Miao Feng.

Project administration: Yang Li.

Resources: Miao Feng, Haoran Si.

Software: Haoran Si.

Supervision: Yang Li.

Validation: Miao Feng, Haoran Si.

Visualization: Junrui Zhang.

Writing – original draft: Miao Feng.

Writing – review & editing: Yang Li.
